# Tree ring phototropism and implications for the rotation of the North China Block

**DOI:** 10.1038/s41598-019-41339-2

**Published:** 2019-03-19

**Authors:** Zikun Jiang, Benpei Liu, Yongdong Wang, Min Huang, Tom Kapitany, Ning Tian, Yong Cao, Yuanzheng Lu, Shenghui Deng

**Affiliations:** 1 Chinese Academy of Geological Sciences, Beijing, 100037 China; 2 State Key Laboratory of Palaeobiology and Stratigraphy, Nanjing Institute of Geology and Palaeontology, and Center for Excellence in Life and Palaeoenvironment, Chinese Academy of Sciences, Nanjing, 210008 China; 3 China University of Geosciences, Beijing, 100083 China; 4 The National Dinosaur Museum, Canberra ACT, 2913 Australia; 5 College of Palaeontology, Shenyang Normal University, Shenyang, 110034 China; 6 Key Laboratory of Palaeomagnetism and Tectonic Reconstruction of Ministry of Land and Resources, Institute of Geomechanics, Chinese Academy of Geological Sciences, Beijing, 100081 China; 7 Research Institute of Petroleum Exploration & Development, PetroChina, Beijing, 100083 China

## Abstract

Trees grow towards the sunlight via a process of phototropism. The trunk phototropism processes are frequently observed in Northern Hemisphere from high latitude to at least the Tropic of Cancer region, and also occur in some *in situ* preserved vertical petrified woods in various geological ages. However, such evidence is still very limited and poorly known in fossil record; and the relationship between tree ring phototropism and rotation of tectonic blocks is unclear. Here we report the eccentricities of living and fossil trees as a proxy to determine geological block rotation at the same latitudes within the North China Block. The dominant eccentricity of living trees is southwest 219° ± 5°. By contrast, standing *in situ* fossil trunks in the Mid-Late Jurassic Tiaojishan Formation and the Late Jurassic Tuchengzi Formation had average eccentricities of 237° and 233.5°, respectively. These differences shed light on the palaeogeographical changes, indicating that the North China Block rotated clockwise from the Late Jurassic to the present day. This result is largely coincident with the palaeomagnetic results, indicating that the North China Block rotated clockwise by 26.5° ± 5.5° since the Middle to Late Jurassic transition.

## Introduction

Light is a key environmental factor that drives many aspects of plant growth and development^1^. Phototropism, the reorientation of growth towards light, is one of the most important adaptive processes^2^. Many results have been acquired for phototropism in a variety of aspects since Charles Darwin^3^ published “The Power of Movement in Plants” e.g.^1,2,4–11^. Most growth ring studies focused on the dendrochronology as well as its utility for palaeoecologic and palaeoclimatic investigations in deep time e.g.^12–33^. However, little attention has been paid to the trunk phototropism represented by asymmetric growth of tree rings in response to a directed light source. In recent years, increased data have been accumulated for such eccentricity phototropism in both living and fossil tree ring observations, especially in some well-preserved individual fossil trunks^34,35^. However, such evidence is still very limited and poorly known in fossil record and the relationship between ring phototropism and rotation of block is undetermined. Here we report our recent systematic field surveys and investigation results on both living trees and *in situ* fossil wood from the North China Block, including 253 living trees from Beijing and Jilin Provinces, and 7 fossil *in situ* trunks from the Jurassic Tiaojishan and Tuchengzi formations in Liaoning and Beijing regions (Supplementary Information S1).

As eccentricity refers to the directional measurement of the longest distance from the pith to the outermost growth ring of an *in situ* tree trunk (southwest direction, when a tree with two largest growing directions), we thus use tree ring eccentricity as a proxy to determine the general block rotation. In addition, our palaeomagnatic data from the Tiaojishan Formation in Beipiao of Liaoning Province also provide support for the fossil data interpretation, indicating that the North China Block had rotated clockwise since the Middle to Late Jurassic transition (Supplementary Information S1).

## Results

### Phototropism in extant tree trunks

**Figure 1:**
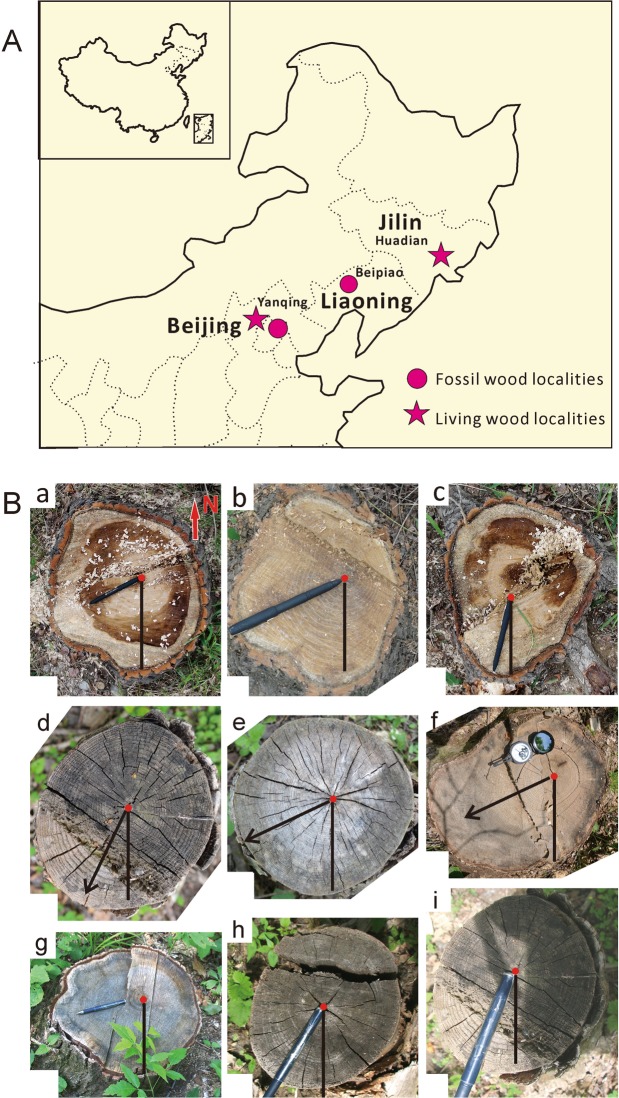
Representative living trees showing eccentricity in Xiangshan of Beijing and Huadian of Jilin Provinces (**A**), Living wood and fossil wood localities in North China region; (**B**) Living trees’ phototropism directions. (a–c) From Xiangshan of Beijing with the eccentricity as 243°, 193°and 245° respectively. (d–i) From Huadian of Jilin Province with eccentricity directions as 210°, 240°, 240°, 202°, 212° and 255° respectively. Red arrow showing the geographical North direction. Black arrows and pens showing the largest growing part direction of wood.

**Figure 2:**
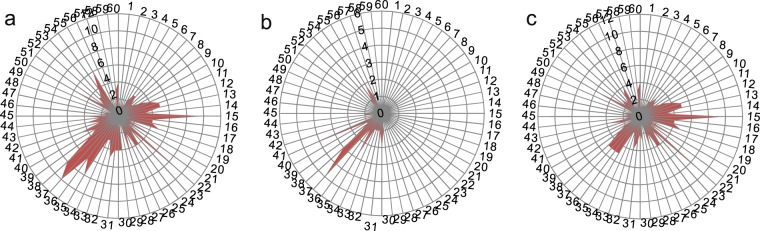
Radar map of living tree data. (**a**) Compilation of all data from localities of Xiangshan and Hongshi; (**b**,**c**) individual radar maps of data from Xiangshan (**b**), and Hongshi (**c**).

The shape, dominant eccentricities and other directions from the pith to the largest growing part (when a tree trunk with two largest growing parts) of living tree trunks were observed within the latitude ranges from 39°59.6′N to 43°15′N in northern China region. We measured 35 trunks at the Wofusi locality in Xiangshan of northern Beijing (39°59′36′′N), and 218 tree trunks at the Hongshi Forestry locality of Huadian, Jilin Province (43°15′52.5′′N) (Fig. 1) (Supplementary Information S2). In each site, the standing and recently fallen trunks were photographed and measured for their eccentricities. The eccentricity data of these living trees examined from Beijing and Jilin Provinces are shown in radar figures (Fig. 2).

The dominant direction of locality in Xiangshan is 222° (Fig. 2b, sector 37 on the circle), which indicates that the trees grow towards the light stimuli. The directions at 180° and 336° might result from two trees that shaded each other (Fig. 2b, sectors 30 and 57). In Honshi locality, the phototropism orientation ranges from 210° to 228° (Fig. 2c, sectors 35–37). The scatter of other orientations around the circle may indicate that the trees in Hongshi locality were influenced by factors other than light (such as gravity and shading). Both data from Xiangshan and Hongshi localities (Fig. 2a) show that the dominant growth direction of eccentricity is southwest, and their average value among the dominant sectors is 219° ± 5°, representing the profound phototropism in Beijing and Jilin Provinces. A secondary peak points directly eastward may result from the influence of other factors, such as gravity in mountain areas, water sources, or shading by other trees.

### Phototropism of *in situ* petrified wood from the Tiaojishan Formation

Three *in situ* standing petrified fossil tree trunks with intact and distinct growth-rings are preserved in the fine-grained sandstone beds of the Middle to Late Jurassic Tiaojishan Formation in Heigou locality, Batuying Town of Beipiao City, Liaoning Province (41.5°N, 120.7°E) (Fig. 1A) (Supplementary Information S1).

**Figure 3:**
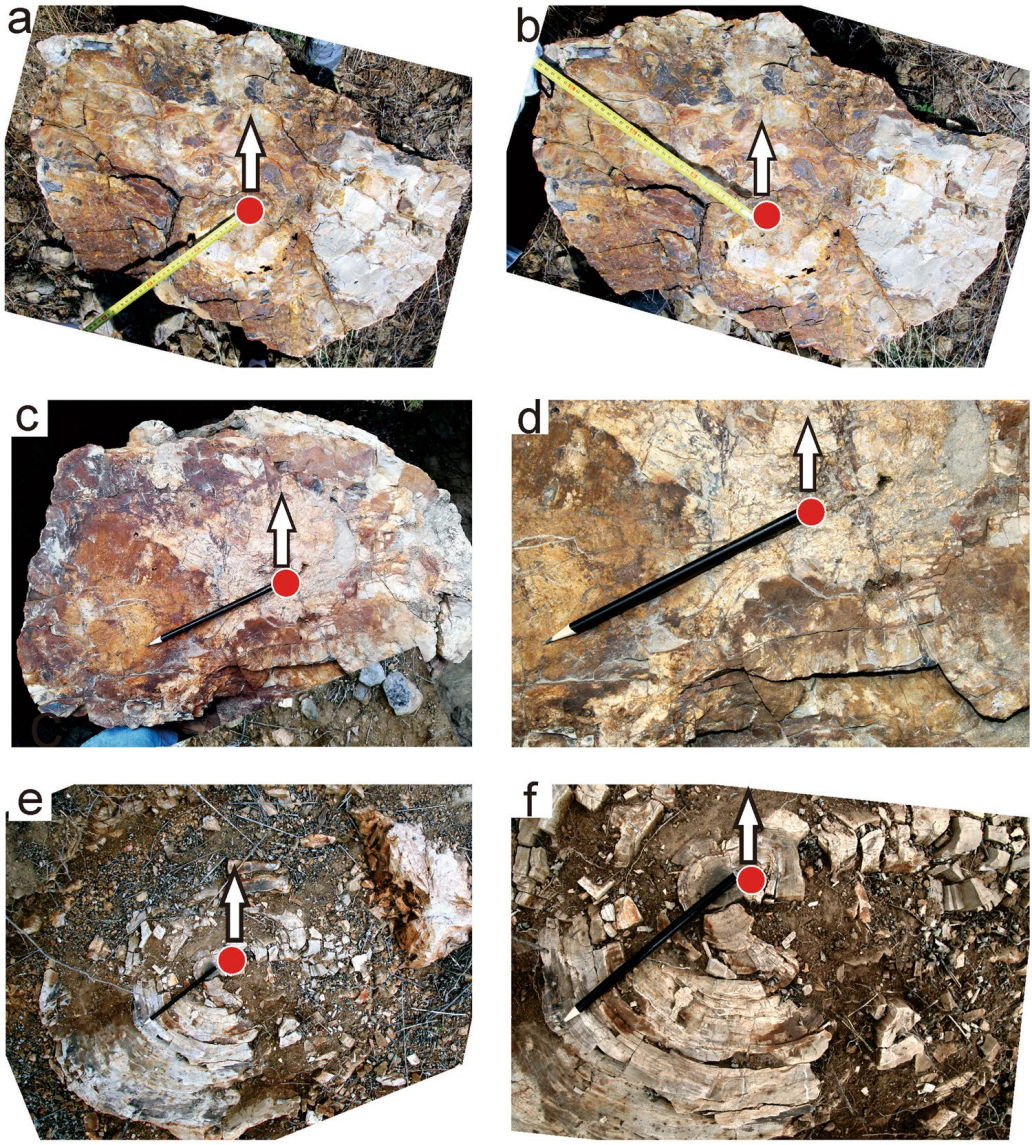
Phototropism in the *in situ* petrified tree trunks from the Tiaojishan Formation of the Late-Middle Jurassic transition in Beipiao of Liaoning Province (**a**,**b**), Petrified trunk No. TJS 1; (**c**,**d**) Fossil wood trunk No. TJS 2; (**e**,**f**) Fossil wood trunk No. TJS 3. Red points indicating piths, black arrows showing the north direction, rulers and pencils direction showing the largest growing part.

The petrified wood trunk No. TJS 1 displays two outermost directions, with the principal eccentricity as 233° (towards the SW) (Fig. 3a), and the other direction as 305° (towards the NW) (Fig. 3b). A potential explanation for this later direction (305°) might be influenced by other disturbing factors, such as shading or other influencing factors (except gravity and water). Fossil wood trunks No. TJS 2 and No. TJS 3 shows an eccentricity of 247° (Fig. 3c,d) and 230° eccentricity (Fig. 3e,f), respectively (Table 1).

**Table 1:** The eccentricity of *in situ* preserved petrified wood in the Tiaojishan and Tuchengzi Formations.

No.	Formation	GPS position	locality	Dominant Eccentricity	The other largest outermost directions
TJS 1	the Tiaojishan Formation	41°27′30″N, 120°47′13″E	Beipiao of western Liaoning province	233°	305°
TJS 2	the Tiaojishan Formation	41°27′30″N, 120°47′12″E	Beipiao of western Liaoning province	247°	No
TJS 3	the Tiaojishan Formation	41°21′30″N, 120°47′12″E	Beipiao of western Liaoning province	230°	No
TCZ 1	the Tuchengzi Formation	40°42′4.1″N, 116°25′15.399″E	Yanqing of Beijing	230°	No
TCZ 2 (modified from reference ^34^—Jiang *et al*., 2014)	the Tuchengzi Formation	40°42′5.301″N, 116°28′15.44″E	Yanqing of Beijing	235°	No
TCZ 3	the Tuchengzi Formation	40°42′5.362″N, 116°23′16.452″E	Yanqing of Beijing	236°	44°
TCZ 4	the Tuchengzi Formation	40°42′6.023″N, 116°23′17.07″E	Yanqing of Beijing	233°	No

Note: TJS = Tiaojishan TCZ = Tuchengzi.

The average eccentricity of these *in situ* standing fossil trunks from the Tiaojishan Formation is 237°. Comparing with the extant phototropism direction and the *in situ* preserved fossil trunks (the difference between 219° ± 5° and 237°), the eccentricity of these petrified trunks shows ca. 13–23° more towards the southwest than that of the living trees.

### Phototropism of *in situ* petrified wood from the Tuchengzi Formation

Four vertical *in situ* petrified tree trunks from the Tuchengzi Formation were examined from the Yanqing Global Geopark in northern Beijing. The Tuchengzi Formation conformably or disconformably overlies the Tiaojishan Formation, and is dated as Late Jurassic to Early Cretaceous in age (approximately 150 Ma)^36^ (Supplementary Information S1).

**Figure 4:**
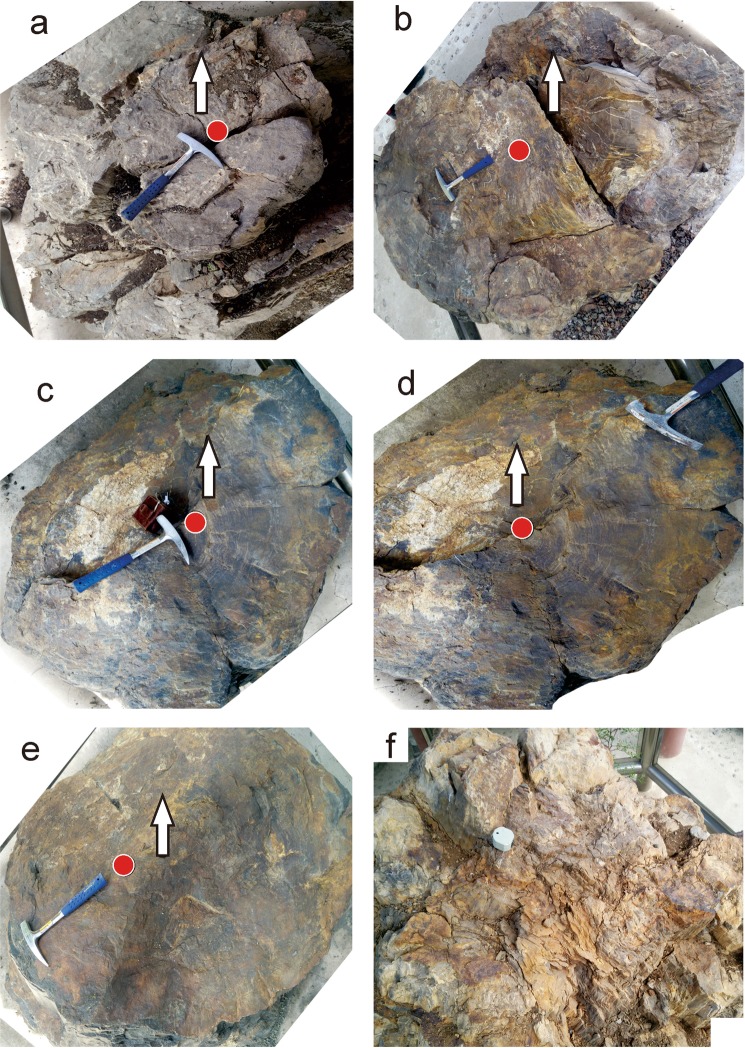
Phototropism in the *in situ* preserved petrified tree trunks from the Late Jurassic Tuchengzi Formation in Yanqing Geopark of Beijing. (**a**) Fossil wood trunk No. TCZ 1; (**b**) Fossil wood trunk No. TCZ 2 (modified from ref.^34^—Jiang *et al*. 2014); (**c**,**d**) Fossil wood trunk No. TCZ 3; (**e**) Fossil wood trunk No. TCZ 4; (**f**) a weathering trunk showing obscure ring features. Red points showing piths of the trunks. Black arrows indicating the North direction. Hammers pointing the largest growing outermost.

The phototropism orientation of fossil wood trunk No. TCZ 1 is 230°(Fig. 4a). Trunk No. TCZ 2 indicates an orientation of 235° (Fig. 4b). Trunk No. TCZ 3 exhibits clear ring elongation in two directions, i.e. 236° (Fig. 4c) and 44° (Fig. 4d). Trunk No. 4 records a phototropism orientation of 233° (Fig. 4e) (Table 1).

The average phototropism direction of these fossil trunks is 233.5° in the Late Jurassic Tuchengzi Formation in the Yanqing of Beijing area, with the same latitude as in Xiangshan locality of living trees in Beijing. Compared with the average phototropism direction recorded in fossilized trees in the Tiaojishan Formation, there is a 3.5° difference in orientation (the difference between 233.5° and 237°). The eccentricity of the wood in the Tuchengzi Formation shows estimates of 9.5–19.5° difference (between 219° ± 5° and 233.5°) more towards the southwest than that of the extant trees. It is thus obvious that the different eccentricities of these two formations might reflect the continuous clockwise rotation of the North China Block.

### Palaeomagnetic results from the Beipiao Basin in the North China Block

**Figure 5:**
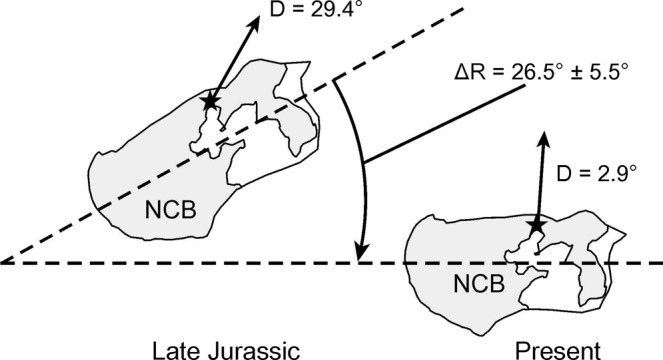
Schematic map illustrating the clockwise rotation of the North China Block from the Late Jurassic and the present day. NCB: North China Block, D: declination angle; ΔR: rotation angle, ★: study region.

About 100 samples for palaeomagnetic analysis were collected from ten sites of the Tiaojishan Formation in Beipiao Basin, approximately 50 meters away from the *in situ* preserved petrified trunk occurrence. The Beipiao Basin is located along the northern margin of the North China Block. The combined mean palaeomagnetic direction of this study and the results from previous study^37^ is Dg = 70.2°, Ig = 75.5°, kg = 12.6, α95 = 9.3° (in geographic coordinates) and Ds = 29.4°, Is = 67.2°, ks = 34.8, α95 = 5.5° (in stratigraphic coordinates). The corresponding paleopole lies at 67.1°N, 175.7°E with A95 = 8.0°. Compared with the present geomagnetic field (PGF) direction (D/I = 2.9°/57.0°), it is suggested that the North China Block has experienced clockwise rotation of 26.5° ± 5.5° since the Late Jurassic (Fig. 5) (Supplementary Information S3). In addition to the previous studies^37^, our palaeomagnetic result provides a more positive support to fossil wood evidence, as the palaeomagnetic sample locations are very close to the *in situ* petrified wood of Tiaojishan Formation in Beipiao, Liaoning Province, which reduced the effect of the local rotation.

## Discussion

In the Northern Hemisphere, trunk phototropism is observed at high latitudes and extends south to at least the Tropic of Cancer (N23°26′)^34^. The magnitude of the phototropic response generally varies from stronger at the poles to weaker at the lower latitudes, finally disappearing near the tropics^34^. Observations of living trees indicate that the eccentricity of growth rings in the Northern Hemisphere trends more southwest than south, possibly due to the fact that trees might preferentially grow faster from after high noon, when the sun is in the southwest sky.

In addition to light, other factors such as gravity, water, shading, wind strength, and chemistry can influence the eccentric tropisms or movements of growth rings^38^. These conclusions are mostly based on observations of trees in mountainous regions, where the combination of phototropism and gravitropism affects the direction of plant growth^38^. To eliminate any potential influence from the above mentioned factors, particularly gravitropism, living trees in flat, unshaded areas were chosen to be the ideal condition to measure the eccentricity. Gravity is the most important influencing factor on tree morphology. In mountainous areas, regardless of whether a tree is located on the north or south slope of an inclined surface, the gravitropism impacts on growing stems. In our studies on living wood, all the tree trunks from Xiangshan of Beijing are located on flat ground without off-centre gravitational interference. Most of the tree trunks at the Hongshi of Jilin Province are on the flat ground, some limited tree trunks are on hills, and thus gravity may disturb some results of eccentricity. Other directions of the tree trunks at Hongshi of Jilin Province are influenced by trees shading by each other.

A reasonable assumption can be made that the growth-ring asymmetry in vertical *in situ* fossil trunks was related almost entirely to sunlight. They were commonly preserved in open and flat environments^39^. The fossil wood in the Tuchengzi Formation in Yanqing of Beijing were generally deposited and buried in a lucastrine-flooding and volcanic ecosystem^40,41^. In the Tiaojishan Formation, *in situ* fossil trunks which were identified as conifer wood were preserved in upland under volcanic explosion which were far away from water sources^39^. The palaeoenvironment of the fossil trunks in the Tuchengzi Formation is also supposed to be forest far away from the lake deposit^39–41^. Obviously, information of geological significance could be thus obtained through an investigation into the shapes and rings of *in situ* fossil tree trunks^42^. Some trees may have two longest outermost growth rings of an *in situ* tree trunk. Therefore, it is crucial to distinguish the nature of these rings to be driven either by the light stimuli or by the other influencing factors.

However, as the mechanisms of phototropism are complex and thus other elements should also be considered for fossil wood trunk eccentricities. For example, petrified wood No. TJS 1 in Beipiao City of Liaoning Province (Fig. 3a,b) and No. TCZ 3 in Yanqing locality of northern Beijing (Fig. 4c,d) exhibits the two largest lengths. We interpret that 233° and 236° are the eccentricity directions (Figs 3a and 4c), and directions 305° and 44° may largely caused by shading or other influencing factors (except gravity and water) (Figs 3b and 4d). It is clear that light greatly contributes to the asymmetry of tree growth, although the degree of that contribution is still unknown. Though steps can be taken to minimize the likelihood that a growing tree was affected by factors other than light, a much larger dataset is required to complete the statistical analysis necessary to reduce signals caused by other influencing factors.

Compared with a large number of fossil fallen tree trunks, *in situ* preserved specimens are scarce. In this study, fieldwork was conducted across most localities of *in situ* fossil wood in China; however, because of weathering (Fig. 4f), transplanting and subsequent damage, a set of valid data were obtained in only three sites, e.g. three specimens were found in Beipiao of western Liaoning Province, and four samples were located in Yanqing of Northern Beijing. Compared with extant trees, a mass of statistical data on fossil wood is needed to determine the specific contribution of light, absent of other factors. Unfortunately, most vertical *in situ* petrified tree trunks have been damaged in the field, thus some of their eccentricities may be unclear.

The Baipiao Basin, from where we collected both fossil wood trunks and palaeomagnetic data, belongs to the North China Block. From the Jurassic to the present day, the Beipiao Basin may have undergone some tectonic movements caused by neighbouring small scale blocks within the North China Block^43–45^. However, such movements are too minor and can be ignored when compared with the block rotation degree of 26.5° ± 5.5°.

The geological age of the Tiaojishan and Tuchengzi formations correspond to the timing of the crustal rotation that occurred in the eastern part of the Yanshan Mountains in North China Plate^36^. The peak period of Yanshan tectonic evolution (165–136 Ma) is characterized by crust-mantle interactions^46^, crustal rotation and subsequent destruction of the craton^47,48^ and the formation of the so-called “Eastern China Plateau”^49^. Thus the rotation of the North China Block inferred by our fossil and living wood data analysis shed new light on the ecosystem response of this profound tectonic movement in East Asia. This is important for understanding the interaction between the various aspects of earth systems during the Mid-Late Jurassic to Early Cretaceous transition, particularly the relationships between tectonic movement and climate adjustments, and the subsequent impact on palaeogeography as well as on fauna migration and evolution.

## Methods

### Field collection and mapping of living wood

All the wood trunks in Xiangshan of Beijing, Huadian of Jilin Provinces were perpendicular to the horizon. Ruler was used to measure the outermost radius. Compass was applied to measure the eccentricity, e.g. the direction (southwest direction, when a tree has two largest growing parts) from the pith to the largest growing part. GPS was used to locate the precise positions. In order to exclude influencing factors other than light stimuli, tree trunks found in flat ground without any shadowing obstacles were selected as the ideal data. If the data were influenced by other factors, it was crucial to eliminate the interference factors in field study. In the common sense, the southwest direction is probably the phototropism direction; other directions are formed by the lateral factors.

We mapped the numbers of living tree data points (253 data) collected in each sector into a radar figure, in which the 360-degree circle was divided evenly into 60 sectors, each sector represents 6 degrees, and the data is arranged by angle in ascending order and sorted into the corresponding sectors.

### Field collection of *in situ* petrified wood

*In situ* preserved petrified wood in two sites were discovered during the fieldwork. Two sites are located in Beipiao of Liaoning for the Tiaojishan Formation and in Beijing for the Tuchengzi Formation. In the Tiaojishan Formation, three stumps were found for measuring the eccentricity. In the Tuchengzi Formation, four stumps were chosen to indicate the phototropism. All the petrified wood trunks are vertical to the bedding plane to ensure *in situ* status. Ruler, compass and GPS units were used when investigating fossil wood in the field. We measured the largest off-centre data of fossil wood, which is from the pith to the largest outermost of the trunk. In the field work, some fossil trunks have two largest growing directions, i.e. No. TJS 1 (233° and 305°) and No TCZ 3 (236° and 44°). The southwest directions (233° and 236°) are thus the direction of eccentricity, while the other directions (305° and 44°) may due to other influencing factors, i.e. shaded with other trees. For the *in situ* fossil trunks preserved in the open and flat environment and far away from the water sources^39–41^, the influencing factor, i.e. gravity and near water sources are excluded.

During the field survey, we emphasized that both living and fossil trees may have a single, non-hollow trunk, and exhibit annual growth cycles. Structural changes caused by disease or damage by insects were not observed. Because the difference of tree species types does not affect heavily on the phototropism of living wood^50,51^, therefore we followed this principle and did not distinguish their taxonomic position. In our study, all the fossil wood trunks are represented by gymnosperms. Living trees in Xiangshan of Beijing are angiosperms, whereas trees in Huadian of Jilin are 80% gymnosperms (conifers), and 20% are angiosperms.

All the growth rings in both living and fossil trees are larger than 30 circles, which means the trees are all older than 30 years. The average height of *in situ* preserved trees in the Tiaojishan Formation is about 25 m^52^.

### Palaeomagnetic data

We collected ten sites (approximately 100 samples) for palaeomagnetic analysis from the Tiaojishan Formation in Beipiao Basin, very close to the *in situ* preserved petrified wood samples. The occurrence of the stratum was measured on the intercalated sandstone layer in volcanic tuff (Supplementary Information S3). The strike and dip of the stratum is 42° and 14°, respectively. In total, material from ten sites were sampled using a gasoline-powered drill, and approximately ten oriented samples were collected from each site.

The samples were cut into cylinders 2.2 cm long for subsequent palaeomagnetic analysis. All samples underwent stepwise thermal demagnetization up to 680 °C that was performed with an ASC TD-48 thermal demagnetizer with an internal residual field of <10 nT. The demagnetization temperature intervals were generally large (40–50 °C) in the low-temperature part and smaller (20–30 °C) at higher temperatures. Remnant magnetizations were measured using a 2G-755R cryogenic magnetometer and a JR-6 spinner magnetometer. All measurements were carried out in a shielded room with residual fields of <300 nT at the Key Laboratory of Palaeomagnetism and Tectonic Reconstruction of the Ministry of Land and Resources, Institute of Geomechanics, Chinese Academy of Geological Science in Beijing. Magnetization directions were determined by principal component analysis^53^ or remagnetisation circle analysis^54^. The average palaeomagnetic direction was counted with Fisher statistics^55^ or the mixed mean of the unit vectors and remagnetisation great circles^56^. The computer program Kirsch developed by Enkin^57^ and PaleoMac developed by Cogné^58^ were used to analyse the palaeomagnetic data.

## Supplementary information

Supplementary information for Tree ring phototropism and implications for the rotation of the North China Block

## Data Availability

Additional data that support the findings of this study are available from the corresponding authors upon request.
