# Supplementary material for: Tree ring phototropism and implications for the rotation of the North China Block
Source: Sci Rep. 2019 Mar 19;9:4856. doi: 10.1038/s41598-019-41339-2 (PMC6425038; doi:10.1038/s41598-019-41339-2)
Supplement: Supplementary file 1 — Supplementary information for Tree ring phototropism and implications for the rotation of the North China Block [file 41598_2019_41339_MOESM1_ESM.doc]

Supplementary Information for

**Tree ring phototropism and implications for the rotation of the North China Block**

Zikun Jiang1,2*, Benpei Liu3, Yongdong Wang2*, Min Huang1, Tom Kapitany4, Ning Tian5, Yong Cao6, Yuanzheng Lu7, Shenghui Deng7

1 Chinese Academy of Geological Sciences, Beijing 100037, China;

2 State Key Laboratory of Palaeobiology and Stratigraphy, Nanjing Institute of Geology and Palaeontology, and Center for Excellence in Life and Paleoenvironment, Chinese Academy of Sciences, Nanjing 210008, China;

3 China University of Geosciences, Beijing 100083, China;

4 The National Dinosaur Museum, Canberra ACT 2913, Australia;

5 College of Palaeontology, Shenyang Normal University, Shenyang 110034, China;

6 Key Laboratory of Palaeomagnetism and Tectonic Reconstruction of Ministry of Land and Resources, Institute of Geomechanics, Chinese Academy of Geological Sciences, Beijing, 100081, China;

7 Research Institute of Petroleum Exploration & Development, PetroChina, Beijing, 100083, China;

Corresponding author name: Zikun Jiang, Yongdong, Wang

Email: [jiangmaomao_925@sina.com](mailto:jiangmaomao_925@sina.com) or [ydwang@nigpas.ac.cn](mailto:ydwang@nigpas.ac.cn).

**This file includes:**

Supplementary information S1to S3

Supplementary information S1

**Stratigraphy and geological ages of the Tiaojishan and Tuchengzi Formations**

The Jurassic to Cretaceous systems are well developed and exposed in western Liaoning and northern Hebei Provinces (including Beijing) ^1, 2^, forming the proper of the North China Plate. The Middle to Late Jurassic *in situ* preserved fossil wood and palaeomagnatic samples in this study were collected and measured in the Tiaojishan Formation in Heigou Locality, Batuying County, western Liaoning Province. The Late Jurassic *in situ* preserved petrified wood were chosen from the Late Jurassic to the Early Cretaceous Tuchengzi Formation in Yanqing Geopark, North Beijing.

The Tiaojishan Formation lies within the Beipiao Basin along the northern margin of the North China Block. Stratigraphically, the fossil wood specimen was preserved in the Tiaojishan Formation (previous known as the Lanqi Formation). The representative type section of the Tiaojishan Formation is designated at Dashuiquanzi to Wubaoshigou villages in Beipiao City, western Liaoning Province ^3^. This formation conformably or unconformably covers the Haifanggou Formation; and unconformably overlies the Tuchengzi Formation (1). The Tiaojishan Formation is usually considered to be Middle Jurassic in age, based on palaeontological assemblages ^3, 4^. Recent isotopic dating of 40Ar/39Ar in volcanic rock revealed, however, a transition between the late Middle Jurassic and the early Late Jurassic ages for the Tiaojishan Formation, corresponding to Callovian - Oxfordian in age ^5, 6^. The age of the Tiaojishan Formation is thus generally regarded as ca. 155 Ma ^7, 8^.

The Tiaojishan Formation is 2000 m thick and is lithologically composed of intermediate lava and pyroclastic rocks, intercalated with basic volcanic rocks and sedimentary deposits ^1, 9^, with plant-bearing beds made ofsedimentary rocks. . These beds contain abundant and well-preserved fossil plants, including leaf foliages, seeds and fruits, permineralized rhizomes and fossil wood ^10^. Many anatomically preserved plant specimens were recently reported from the Tiaojishan Formation, including fern rhizomes *Ashicaulis* ^11-14^, cycad stem *Lioxylon*^15^, conifers *Araucariopitys*, *Pinoxylon*, *Sciadopityoxylon Xenoxylon* and Ginkgoales *Ginkgoxylon*^10, 16, 17^. Besides plant fossils, other diverse fossil groups were reported in the Tiaojishan Formation, i.e. conchostracans, ostracods, insets, bivalves and amount of vertebrate fossils^18-22^.

Our specimens were preserved in Heigou of Batuying County in Beipiao City, western Liaoning Province. The fossil bearing bed is made of sedimentary rocks without lacustrine deposits. All the in situ preserved fossil trunks are vertical to the bedding plane. The Tuchengzi Formation occurs as a set of characteristic continental sediments in northern Hebei and western Liaoning Provinces. It is formed in a period transitional from Jurassic-Cretaceous boundary in China^23^, distributed in central part of Europe-Asia continent, and represented by light red and variegated terrigenous classic rocks, containing various fossil flora and faunagroups ^2^.

The sediments of the Tuchengzi Formation in both northern Hebei and western Liaoning are mainly composed of purplish red, grayish purple, grayish white, grayish green and yellowish green terrigenous clastics. Lithologically, they are represented by polygenetic conglomerate, pebbly coarse-grained sandstone, various grained sandstone, siltstone, silty mudstone, and pelitic shale. The Tuchengzi Formation is stratigraphically equivalent to each other in northern Hebei and western Liaoning, and is underlain by the same eruptive rocks of the Middle to Late Jurassic Tiaojishan Formation^2^. In between these two formations, there appears a sedimentary hiatus, which is regarded as a parallel unconformity, or a sedimentary overlapping in margins of a basin, or a trachy discontinuity caused by uneven accumulation of volcanic lava flows ^24^.

The Tuchengzi Formation is formed generally under the arid and hot climate conditions in this region,characterized by purplish red, grayish purple and brownish red clastic sediments poor in fossils. However, a biota represented by *Chaoyangsaurus*, *Pseudograpta* and *Catacella*, the so-called Tuchengzi Biota, occurred and developed regionally in the Yanshan-Liaoning area ^2^. This biota is many composed of dinosaurs, fishes, conchostracans, ostracods, insets, bivalves, plants (including fossil wood) and sporo-pollens, with more than 132 genera and 290 species ^2^. Vertical fossil trunks are preserved in the greyish-green tuffaceous mudstone of the Upper Jurassic bed in Yanqing of Beijing ^24^.

The stratotype of the Tuchengzi Formation is designated in the Jijiawopu-Liushugou-Baijiawopu section in Batuyingzi Town of Beipiao City; with the auxiliary stratotype section exposed from Majiagou-Hujiawopu to Qianfodong in Beisijiazi Town of Chaoyang City, western Liaoning Province ^2^ .

Recent investigation shows that the age of the Tuchengzi Formation is Late Jurassic to the Early Cretaceous, approximately 150 Ma ^23^. Our fossil wood specimens are preserved in Xiadelongwan village of Qianjiadian Town in Yanqing County of Beijing City The petrified wood is preserved in the lower part of the Tuchengzi Formation, which is formed in the Late Jurassic age ^25^.

**References**

1. Xu, K. et al. Jurassic in Northern China VII. Northeast Stratigraphic Division. (Petroleum Industry Press, Beijng) pp1–261(2003).
2. Wang, W. L. et al. *Standard sections of Tuchengzi Stage and Yixian Stage and their stratigraphy, palaeontology and Tectonic-Volcanic actions*.(Geological Publishing House, Beijing) pp514 (2004).
3. Wang, W. L. et al. *Mesozoic stratigraphy and palaeontology of Western Liaoning, vol 1*. (in Chinese with English abstract). (Geological Publishing House, Beijing) (1989).
4. Zhang, W. & Zheng, S. L. Early Mesozoic fossil plants in western Liaoning, northeast China (in Chinese). *Mesozoic Stratigraphy and Palaeontology of Western Liaoning (3)* eds. Yu X-H et al. (Geological Publishing House, Beijing) pp 239–338 (1987) (in Chinese).
5. Chang, S., Zhang, H. C., Renne, P. R. & Fang, Y. High-precision 40Ar/39Ar age constraints on the basal Lanqi Formation and its implications for the origin of angiosperm plants. *Earth and Planetary Science Letters* 279, 1–10 (2009).
6. Zhang, H., Wang, M. X., Liu, X. M. Constraints on the upper boundary age of the Tiaojishan Formation volcanic rocks in West Liaoning-North Hebei by LA-LCP-MS dating. *Science Bulletin* 58, 3574–3584 (2008).
7. Pei, J. L. et al. A paleomagnetic study from the Late Jurassic volcanics (155 Ma), North China: implications for the width of Mongol–Okhotsk Ocean. *Tectonophysics* 510, 370–380 (2011).
8. Ren, Q. et al. Further paleomagnetic results from the ~155 Ma Tiaojishan Formation, Yanshan Belt, North China, and their implications for the tectonic evolution of the Mongol–Okhotsk suture. *Gondwana Res.* **35**, 180–191 (2016).
9. Jiang, B. Y., Yao, X. G., Niu, Y. Z., Rao, X. & Li, Q. J. Outline of the Jurassic and Cretaceous systems in western Liaoning. (University of Science and Technology of China Press, Hefei) pp 1–84 (2010) (in English, with Chinese translation).
10. Wang, Y. D., Saiki, K., Zhang, W. & Zheng, S. L. Biodiversity and palaeoclimate of the Middle Jurassic floras from the Tiaojishan Formation in western Liaoning, China. *Prog*. *in Natu*. *Sci*. 16, 222–230 (2006).
11. Tian, N., Wang, Y. D., Zhang, W., Jiang, Z. K. & Dilcher, D. L, *Ashicaulis beipiaoensis* sp. nov., a new Osmundaceous fern species from the Middle Jurassic of Liaoning Province, Northeastern China. *International Journal of Plant Sciences* 174, 328–339 (2013).
12. Tian, N., Wang, Y. D., Zhang, W. & Jiang, Z. K. A new structurally preserved fern rhizome of Osmundaceae (Filicales) Ashicaulis wangii sp. nov. from the Jurassic of Western Liaoning and its significances for palaeobiogeography and evolution. *Science China, Earth Sciences* 57, 671–681 (2014a).
13. Tian, N. et al. A specialized new species of *Ashicaulis* (Osmundaceae, Filicales) from the Jurassic of Liaoning, NE China. *Journal of Plant Research* 127, 209–219 (2014b).
14. Tian, N., Wang, Y. D., Dong, M., Li, L. Q. & Jiang, Z. K. A systematic overview of fossil osmundalean ferns in China: Diversity variation, distribution pattern, and evolutionary implications. *Palaeoworld* 25, 149–169(2016).
15. Zhang, W., Wang, Y. D., Saiki, K., Li, N. & Zheng, S. L. A structurally preserved Cycad-like stem, *Lioxylon* gen. et sp. nov., from the Jurassic in western Liaoning, China. *Progress in Natural Science* 26, (Special issue), 236–248 (2006).
16. Jiang, Z. K., Wang, Y. D., Zheng, S. L. & Zhang, W. Tian N Occurrence of *Sciadopitys*-like fossil wood (conifer) in the Jurassic of western Liaoning and its evolutionary implications. *Chinese Science Bulletin* 57, 569–572 (2012).
17. Jiang, Z. K. et al. A Jirassic wood providing insights into the earliest step in Ginkgo wood evolution.. *Scientific Reports* 6, 38191 (2016).
18. Duan, Y., Zheng, S. L., Hu, D. Y., Zhang, L. J., Wang, W. L. Preliminary report on Middle Jurassic strata and fossils from Linglongta area of Jianchang, Liaoning. *Global Geology* **28**, 143-147 (2009).
19. Hu, D. Y., Hou, L. H., Zhang, L. J. & Xu, X. A pre-*Archaeopteryx* troodontid theropod from China with long feathers on the metatarsus. *Nature* 1-4 (2009).
20. Lü, J. C. A new non-pterodactyloid *pterosaur* from Qinglong County, Hebei Province of China. *Acta Geologica Sinica* **83**, 189-199 (2009).
21. Lü, J. C. & Fucha, X. H. A new *pterosaur* (*Pterosauria*) from Middle Jurassic Tiaojishan Formation of western Liaoning, China. *Global Geology* **13**, 113-118(2011).
22. Luo, Z. X., Yuan, C. X., Meng, Q. J. & Ji, Q. A Jurassic eutherian mammal and divergence of marsupials and placentals. *Nature* **476**, 442-445 (2011).
23. Xu, H., Liu, Y. Q., Kuang, H. W. & Peng, N. Ages of the Tuchengzi Formation in northern China and the terrestrial Jurassic-Cretaceous boundary in China. *Earth Sciences Frontiers* 21, 203-215 (2014).
24. Jiang, Z. K. et al. The Phototropism of Jurassic petrified wood in North China Plate. *Acta Geological Sinica* **88**, 1352-1355 (2014).
25. Jiao, R. C., Wang, R. R., Zhang, S. Y. Occurrence strata and formation age of petrified wood in Qianjiadian Area of Yanqing County. *Urban Geology* 11, 56-59 (2016).

Supplementary information S2

Result of living trees’ eccentricity in Jilin and Beijing

| Spot No. in Xiangshan, Beijing | Data (°) | |
| --- | --- | --- |
| 1 | 221 |  |
| 2 | 210 |  |
| 3 | 270 |  |
| 4 | 330 |  |
| 5 | 320 |  |
| 6 | 243 |  |
| 7 | 193 |  |
| 8 | 220 |  |
| 9 | 192 | 135 |
| 10 | 233 |  |
| 11 | 213 |  |
| 12 | 172 | 352 |
| 13 | 162 |  |
| 14 | 220 |  |
| 15 | 225 |  |
| 16 | 217 |  |
| 17 | 335 |  |
| 18 | 205 |  |
| 19 | 200 |  |
| 20 | 225 |  |
| 21 | 330 |  |
| 22 | 180 |  |
| 23 | 185 |  |
| 24 | 245 | 175 |
| 25 | 235 |  |
| 26 | 245 |  |
| 27 | 220 |  |
| 28 | 175 |  |
| 29 | 263 |  |
| 30 | 245 |  |
| 31 | 225 |  |
| 32 | 205 |  |
| 33 | 313 |  |
| 34 | 243 |  |
| 35 | 35 |  |
|  |  |  |
| Spot No. in Maoshan  Tree Farm in Huadian Data (°)  Jilin Province | | |
| 1 | 120 |  |
| 2 | 160 |  |
| 3 | 170 |  |
| 4 | 230 |  |
| 5 | 300 |  |
| 6 | 320 |  |
| 7 | 70 |  |
| 8 | 90 |  |
| 9 | 130 |  |
| 10 | 210 |  |
| 11 | 90 |  |
| 12 | 130 |  |
| 13 | 220 |  |
| 14 | 30 |  |
| 15 | 215 |  |
| 16 | 95 |  |
| 17 | 75 |  |
| 18 | 210 |  |
| 19 | 315 | 235 |
| 20 | 75 |  |
| 21 | 0 |  |
| 22 | 65 |  |
| 23 | 178 |  |
| 24 | 265 |  |
| 25 | 130 |  |
| 26 | 15 |  |
| 27 | 350 |  |
| 28 | 65 |  |
| 29 | / |  |
| 30 | 55 |  |
| 31 | / |  |
| 32 | 40 |  |
| 33 | 47 |  |
| 34 | 90 |  |
| 35 | 78 |  |
| 36 | 300 |  |
| 37 | 74 |  |
| 38 | 327 |  |
| 39 | / |  |
| 40 | 170 |  |
| 41 | 50 |  |
| 42 | 40 |  |
| 43 | 55 |  |
| 44 | / |  |
| 45 | 58 |  |
| 46 | 135 |  |
| 47 | 105 |  |
| 48 | 130 |  |
| 49 | 100 |  |
| 50 | 73 |  |
| 51 | 84 |  |
| 52 | 90 |  |
| 53 | 95 |  |
| 54 | 107 |  |
| 55 | 65 |  |
| 56 | 220 |  |
| 57 | 65 |  |
| 58 | 85 |  |
| 59 | 88 |  |
| 60 | 45 |  |
| 61 | 68 |  |
| 62 | / |  |
| 63 | / |  |
| 64 | 130 |  |
| 65 | 355 |  |
| 66 | 210 |  |
| 67 | 258 |  |
| 68 | 100 |  |
| 69 | 100 |  |
| 70 | 205 |  |
| 71 | 310 |  |
| 72 | 130 |  |
| 73 | 85 |  |
| 74 | 115 |  |
| 75 | / |  |
| 76 | / |  |
| 77 | 340 |  |
| 78 | 295 |  |
| 79 | 330 |  |
| 80 | 150 |  |
| 81 | 158 |  |
| 82 | / |  |
| 83 | / |  |
| 84 | 120 |  |
| 85 | / |  |
| 86 | / |  |
| 87 | 95 |  |
| 88 | 145 |  |
| 89 | / |  |
| 90 | / |  |
| 91 | 115 |  |
| 92 | 160 |  |
| 93 | / |  |
| 94 | 68 |  |
| 95 | 25 |  |
| 96 | / |  |
| 97 | 90 |  |
| 98 | 85 |  |
| 99 | / |  |
| 100 | / |  |
| 101 | 142 |  |
| 102 | 140 |  |
| 103 | / |  |
| 104 | / |  |
| 105 | 115 |  |
| 106 | 40 |  |
| 107 | 225 |  |
| 108 | 210 |  |
| 109 | 312 |  |
| 110 | 330 |  |
| 111 | / |  |
| 112 | 150 |  |
| 113 | 107 |  |
| 114 | 120 |  |
| 115 | 325 |  |
| 116 | 315 |  |
| 117 | / |  |
| 118 | 130 |  |
| 119 | 103 |  |
| 120 | 132 |  |
| 121 | 345 |  |
| 122 | 155 |  |
| 123 | 130 |  |
| 124 | 355 |  |
| 125 | / |  |
| 126 | 181 |  |
| 127 | 225 |  |
| 128 | 69 |  |
| 129 | 288 |  |
| 130 | 228 |  |
| 131 | 212 |  |
| 132 | 237 |  |
| 133 | 202 |  |
| 134 | 212 |  |
| 135 | 282 |  |
| 136 | / |  |
| 137 | 202 |  |
| 138 | 275 |  |
| 139 | 224 |  |
| 140 | 152 |  |
| 141 | 127 |  |
| 142 | 182 |  |
| 143 | 122 |  |
| 144 | 282 |  |
| 145 | 197 |  |
| 146 | 99 |  |
| 147 | / |  |
| 148 | 32 |  |
| 149 | 302 |  |
| Spot No. in Hongshi Tree Data (°)  Farm in Huadian, Jilin | | |
| 1 | 247 |  |
| 2 | / |  |
| 3 | / |  |
| 4 | 215 |  |
| 5 | 70 |  |
| 6 | / |  |
| 7 | 190 |  |
| 8 | 310 |  |
| 9 | 220 |  |
| 10 | 140 |  |
| 11 | 300 |  |
| 12 | 250 |  |
| 13 | 105 |  |
| 14 | 162 |  |
| 15 | 320 |  |
| 16 | 80 |  |
| 17 | 305 |  |
| 18 | / |  |
| 19 | 120 |  |
| 20 | / |  |
| 21 | 85 |  |
| 22 | 270 |  |
| 23 | / |  |
| 24 | / |  |
| 25 | 295 |  |
| 26 | 20 |  |
| 27 | / |  |
| 28 | / |  |
| 29 | / |  |
| 30 | / |  |
| 31 | 140 |  |
| 32 | / |  |
| 33 | / |  |
| 34 | 215 |  |
| 35 | 190 |  |
| 36 | / |  |
| 37 | 270 |  |
| 38 | 0 |  |
| 39 | / |  |
| 40 | 195 |  |
| 41 | / |  |
| 42 | / |  |
| 43 | 190 |  |
| 44 | 115 |  |
| 45 | 170 |  |
| 46 | / |  |
| 47 | 15 |  |
| 48 | 145 |  |
| 49 | 225 |  |
| 50 | 300 |  |
| 51 | 82 |  |
| 52 | 222 |  |
| 53 | 257 |  |
| 54 | 282 |  |
| 55 | 322 | 257 |
| 56 | 232 |  |
| 57 | 182 |  |
| 58 | 222 |  |
| 59 | 112 |  |
| 60 | 92 |  |
| 61 | 255 |  |
| 62 | 127 |  |
| 63 | 190 |  |
| 64 | 233 |  |
| 65 | / |  |
| 66 | 50 |  |
| 67 | 35 |  |
| 68 | 260 |  |
| 69 | 350 |  |

Supplementary information S3

**Palaeomagnetic results and data analysis**

1. **Palaeomagnetic results**

Zijderveld plots for the representative samples from the Tiaojishan Formation Fig S1 are shown in Fig. S2. In most samples, a lower-temperature component (LTC) and a higher-temperature component (HTC) could be isolated. The LTC was closed to the present geomagnetic field (PGF) direction (PGF: D/I=2.9°/57.0°; LTC: D/I=351.3°/59.7° with a_95_=2.8° in geographic coordinates; sampling site: 41.5°N, 120.7°E) (Fig. S3). The HTC of most samples decays towards the origin, and the magnetization directions could be analysed using principal component analysis^1^ (Fig. S2a-S2e). In addition, the HTC of some of the samples are distributed along a great circle in an equal-area plot, and thus, the magnetization directions could be analysed by remagnetization circle analysis^2^ (Fig. S2f-S2g). The sample mean of the palaeomagnetic directions analysed by remagnetization circles analysis was calculated using the mixed mean of the principal component and the remagnetization great circles ^3^.

**
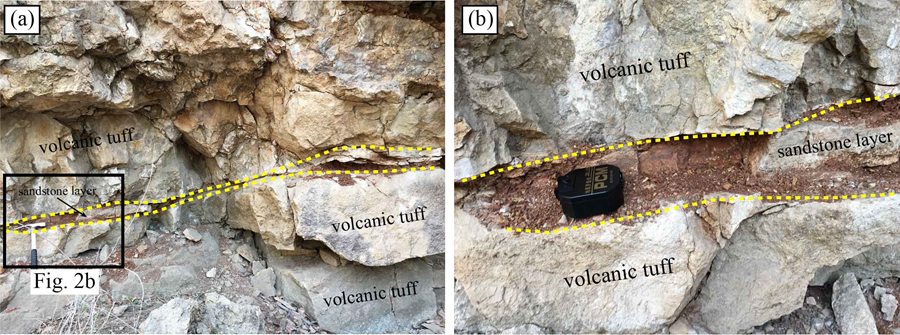
**

Fig. S1. Lithological characte of a field outcrop in the Tiaojishan Formation


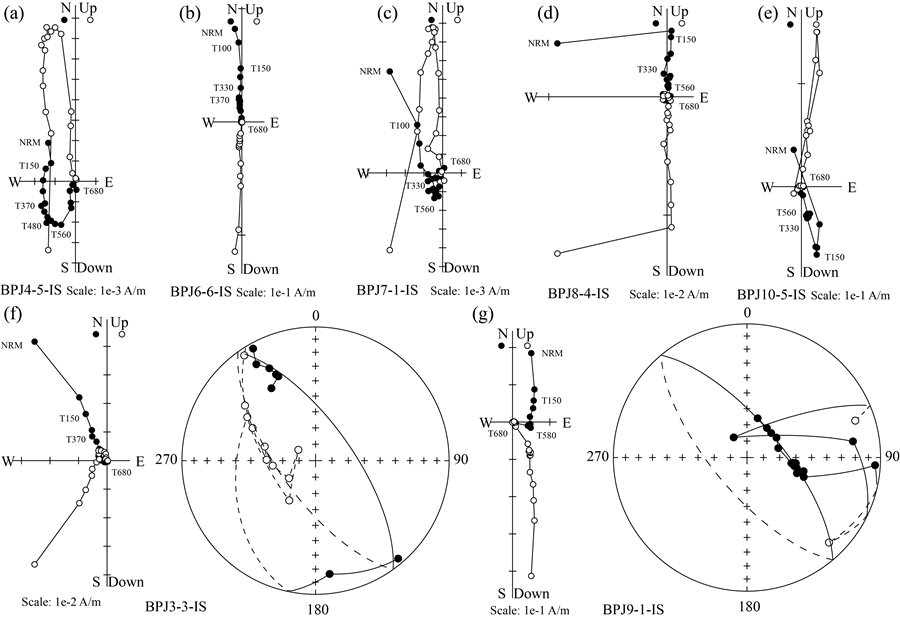


Fig. S2. (a-e) Orthogonal demagnetization diagrams of the coordinates of *in situ* representative samples. (f-g) Orthogonal demagnetization diagrams and equal-area plots of representative samples determined by remagnetization circle analysis. The demagnetization steps are in °C in all plots. NRM: natural remnant magnetization.


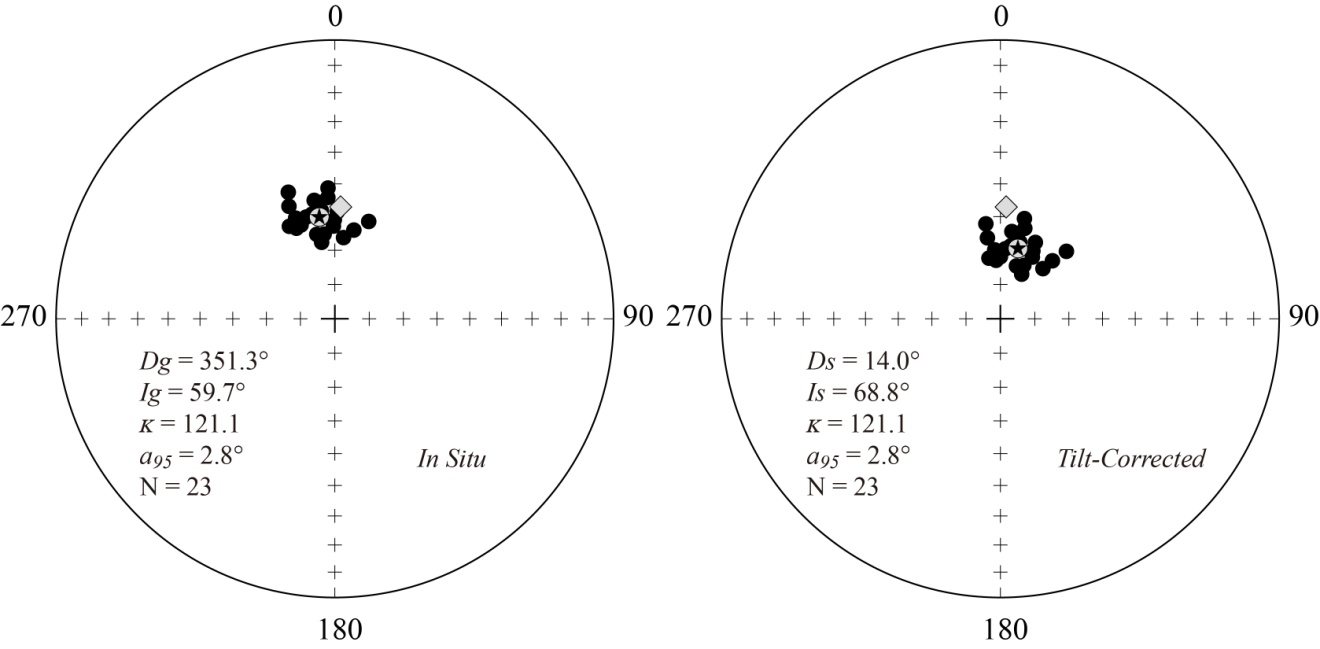


Fig. S3. Equal-area projections of the LTC directions from the Tiaojishan Formation samples. The stars indicate the overall-mean direction of the samples. The rhombuses indicate the PGF direction.

The site-mean directions of the Tiaojishan Formation are listed in Table 1 and illustrated in Fig. S4. The mean direction of 10 sites is *Dg* = 18.5°, *Ig* = 66.0°, *kg* = 32.9, *α_95_* = 8.6° (in geographic coordinates) and *Ds* = 46.2°, *Is* = 68.0°, *ks* = 31.5, *α_95_* = 8.7° (in stratigraphic coordinates). The corresponding palaeomagnetic pole lies at 56.8°N, 174.1°E with A_95_ = 11.9°. The palaeomagnetic directions passed the reversal test with a C classification at the 95% confidence level (r = 12.2° <rc = 16.8°) ^4^. We tested the angular dispersion of the virtual geomagnetic poles (VGPs) and obtained a VGP scatter of 18.3°, which falls within the limit (17.8°–23.7°) of the predicted VGP ranges for the interval from 110 to 195 Ma^5^. This finding suggests that the mean direction of the palaeomagnetic data from the Tiaojishan Formation volcanic tuff in this study may fully average out the secular variation of the earth field.


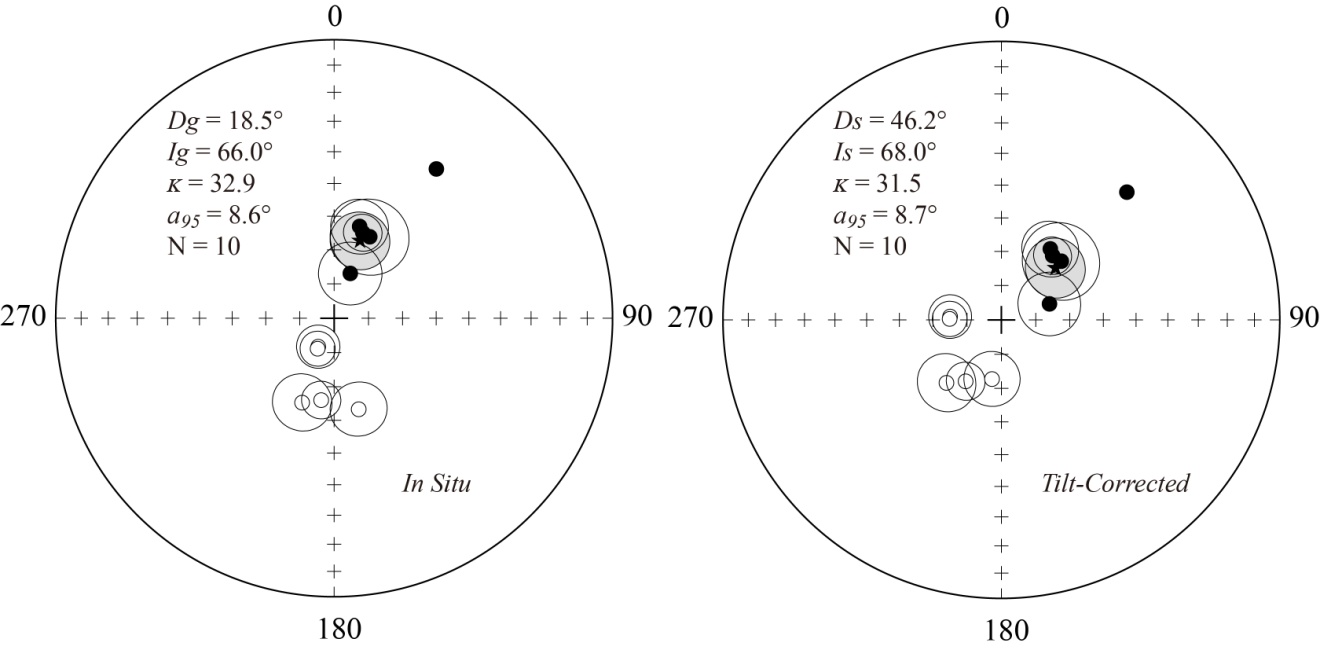


Fig. S4. Equal-area projections of site-mean directions of the Tiaojishan Formation from the Beipiao Basin in the North China Block. Solid/open symbols represent downward/upward inclinations; stars show the overall mean direction of 10 sites.

**2. The rotation of the North China Block since the Late Jurassic**

Palaeomagnetic data from the Tiaojishan Formation in the Beipiao Basin average out the palaeosecular variation and passed reversal tests^6^. Because this palaeomagnetic data are from a similar area and are in good agreement with the findings of Ren et al. (2016), the HTC directions have been combined from both studies to provide a more robust palaeomagnetic pole. The combined mean palaeomagnetic direction is *Dg* = 70.2°, *Ig* = 75.5°, *kg* = 12.6, *α_95_* = 9.3° (in geographic coordinates) and *Ds* = 29.4°, *Is* = 67.2°, *ks* = 34.8, *α_95_* = 5.5° (in stratigraphic coordinates) for the Tiaojishan Formation (Fig. S5). The corresponding palaeopole lies at 67.1°N, 175.7°E with A_95_ = 8.0°. The VGP scatter of the combined palaeomagnetic result is 19.6°, which falls within the range (17.8°–23.7°) of the predicted VGP scatter for the interval from 110 to 195 Ma^5^. This suggests that the overall mean direction of the HTC from both studies (This study and Ren et al., 2016) in the Beipiao area averages out the secular variation. In addition, the combined palaeomagnetic direction passed the reversal test with a C classification at the 95% confidence level (r = 11.0° <rc = 12.4°) ^4^. Hence, the palaeomagnetic direction can be thought to represent the primary magnetization, and it is suitable for a tectonic reconstruction of the North China Block. Compared with the PGF direction (D/I= 2.9°/57.0°), we suggest that the North China Block rotated clockwise by 26.5° ± 5.5° since the Late Jurassic.


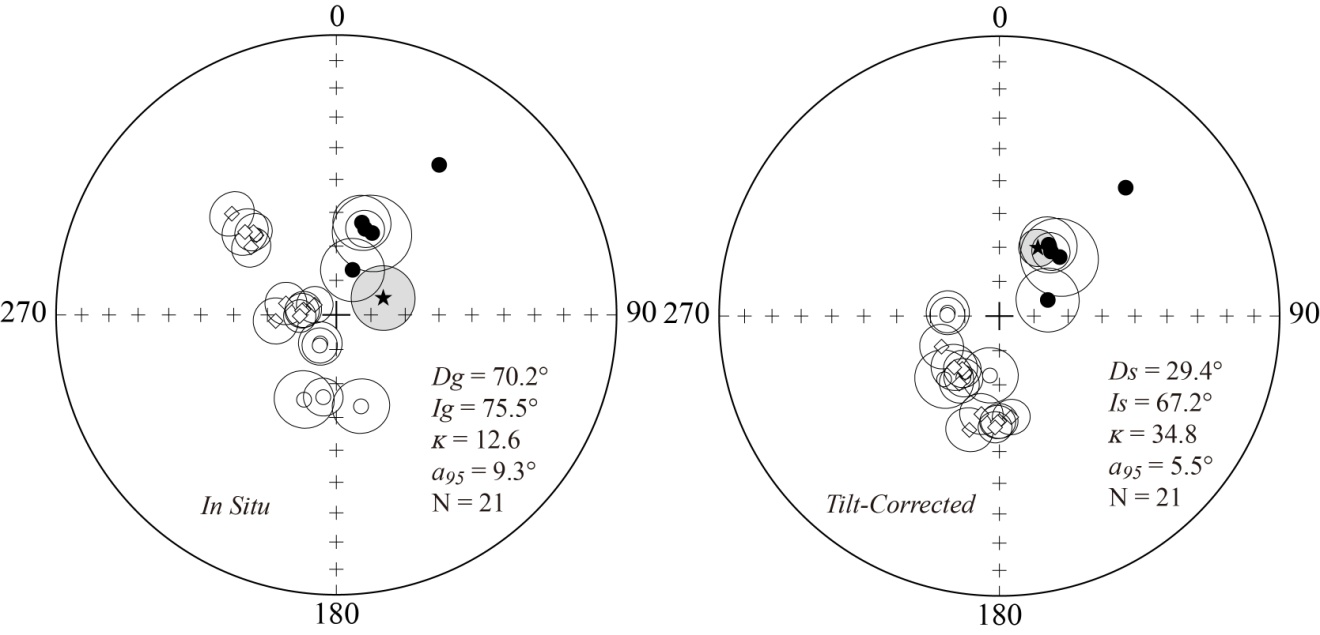


Fig. S5. Equal-area projections of the site-mean directions combined from this study and Ren et al. (2016) for the Tiaojishan Formation in the Beipiao area. Solid/open symbols represent downward/upward inclinations; stars show the overall mean direction of 21 sites.

**References**

1. Kirschvink, J. L. The least-squares line and plane and the analysis of paleomagnetic data. *Geophys*. *J*. *Roy*. *Astron*. *Soci*. **62**, 699–718(1980).
2. Halls H-C The use of converging remagnetization circles in paleomagnetism. *Phys*. *Earth*. *Planet*. *Int*. **16**, 1-11 (1978).
3. McFadden, P. L. & McElhinny, M. W. The combined analysis of remagnetization circles and direct observations in palaeomagnetism. *Earth*. *Planet*. *Sci*. *Lett*. **87**, 161-172 (1988).
4. McFadden, P. L. & McElhinny, M. W. Classification of the reversal test in paleomagnetism. *Geophys*. *J. Int*. **103**, 725–729 (1990).
5. McFadden, P., Merrill, R., McElhinny, M. & Lee, S. Reversals of the Earth's magneticfield and temporal variations of the dynamo families. *J*. *Geophys*. *Res*. 96, 3923–3933 (1991).
6. Ren, Q. et al. Further paleomagnetic results from the ~155 Ma Tiaojishan Formation, Yanshan Belt, North China, and their implications for the tectonicevolution of the Mongol–Okhotsk suture. *Gondwana Res.* **35**, 180–191 (2016).
